# Cost-effectiveness analysis of diagnostic strategies for COVID-19 in Iran

**DOI:** 10.1186/s12913-023-09868-9

**Published:** 2023-08-14

**Authors:** F Rahmanzadeh, N Malekpour, A Faramarzi, H Yusefzadeh

**Affiliations:** 1 Student Research Committee, Urmia University of Medical Sciences, Urmia, Iran; 2 Health Education Department, Urmia University of Medical Sciences, Health deputy, Urmia, Iran; 3 Department of Health Economics and Management, School of Public Health, Urmia University of Medical Sciences, Urmia, Iran

**Keywords:** Chest CT, Cost effectiveness analysis, COVID-19, IgM and IgG antibodies, Polymerase chain reaction (PCR)

## Abstract

**Background:**

Since 2020, COVID-19 has become a global public health issue and has caused problems worldwide. This infection can lead to a fever and respiratory problems. Asymptomatic carriers of the virus are a significant part of the spread of the disease, so early screening and diagnosis of suspected cases of COVID-19 are essential. Generally, standard diagnostic methods include lung imaging (CT), polymerase chain reaction (PCR), and corona antibody (IgM&IgG) testing. However, the costs of the above tests for the healthcare system cannot be ignored, and evaluating the incremental costs against the additional benefit is necessary. Therefore, this study aimed to determine the cost-effectiveness of diagnostic methods for COVID-19 patients.

**Materials and methods:**

In this research, an economic evaluation analysis was conducted to reveal the cost-effectiveness of the diagnostic strategies for COVID-19 from the service provider’s perspective. Basic information about the costs of CT, serology (IgG&IgM), and molecular (PCR) tests were collected from the Ministry of Health of Iran. The effectiveness data were calculated according to the sensitivity and specificity of the diagnostic tests for COVID-19. In this study, the incremental cost-effectiveness ratio (ICER) of the diagnostic strategies for COVID-19 was estimated, and the most cost-effective diagnostic strategy was determined. In calculating ICER and analyzing the sensitivity of the results, Treeage software was used.

**Results:**

According to the calculated incremental effectiveness cost ratio for scenarios with 5, 10, and 50% prevalence of COVID-19 and according to the threshold defined by the World Health Organization, in the study, PCR, PCR, and IgG&IgM strategies are the most cost-effective diagnostic methods of the corona. Also, the results were not sensitive to the desired parameters based on the results of one-way sensitivity analysis.

**Conclusion:**

Nowadays there are various tests with different levels of accuracy in the diagnosis of COVID-19. In general, PCR tests are more cost-effective for low prevalence of Covid-19, while IgM&IgG tests are more cost-effective for high estimated prevalence. The results of this research can help policymakers and health system managers to validate the most accurate diagnostic method for COVID-19, considering the prevalence of the disease.

## Introduction

COVID-19 is a new viral pneumonia that emerged in Wuhan, China, in late 2019 and has spread rapidly around the world, causing a global pandemic [1]. It is more contagious than previous coronaviruses [2] and has no specific treatment or vaccine [3]. Early diagnosis and isolation of patients are crucial to control the outbreak [4, 5]. Many cities in China imposed travel restrictions to limit the spread of the virus [6].

Since the initial outbreak, asymptomatic carriers of the virus and related infections have been reported in several studies [7, 8]. Following these findings, the asymptomatic and preclinical infection may be a significant part of the spread of the disease [4]. Here, containing and controlling the progressive epidemic can also be challenging. Additionally, limited diagnostic tests cause underreporting of cases that require attention to diagnostic tests’ reliability; incorrect testing due to deficiencies in case identification and premature discharges may lead to non-quarantine contaminants [9].

To prevent the disease from spreading, it is vital to use accurate diagnostic tests to detect and isolate infected patients. However, the emergence of new variants and the diversity of methods to identify COVID-19 pose many diagnostic challenges.

The combination test of corona antibodies (IgM&IgG) is a method for diagnosing COVID-19, which is performed serologically and based on the ELISA method. WHO approves this method as an additional tool to check a person’s history of infection with the COVID-19 virus. Currently, this test is used as a standard method to check the history of infection of people with the coronavirus due to its low price, easy access, short duration of testing, convenient sampling, and no need for highly specialized laboratory equipment.

The diagnosis of COVID-19, chest CT plays a central role in staging the disease and evaluating treatment effectiveness. However, the Polymerase Chain Reaction (PCR) is still the primary test for diagnosing COVID-19 [10, 11]. However, it is limited to virus detection and has significant limitations, including the lack of identification of the disease stage [12]. Currently, PCR is recommended as the standard for confirmation of COVID-19 [9]. The accuracy of PCR has been questioned in recent analyses, which have estimated the sensitivity of PCR to be 30–70%. Previously, healthcare professionals have presented evidence that the sensitivity of PCR is about 30-50%, and some experts require two negative PCR results as the definitive exclusion criteria for the diagnosis and discharge of people with COVID-19 [13]. Recent studies claim that chest CT, being more expensive, may be able to detect the disease with higher sensitivity compared to serological and molecular methods [14].

Only by using the optimal diagnostic tools available can health professionals increase the detection rate through additional testing. However, the cost of diagnostic tests to the healthcare system should be addressed, and it is necessary to evaluate the incremental costs against the incremental benefits [15]. Based on the available reviews, such an evaluation still needs to be created. Meanwhile, healthcare decision-makers must consider the health benefits of reducing infections when considering the effectiveness and costs of interventions for infectious diseases. For COVID-19, this indicates that the impact of interventions on asymptomatic and preclinical transmission should also be considered. The present study analyzed the cost-effectiveness of PCR, CT, and corona antibody combination (IgM&IgG) testing for diagnosing people with COVID-19. Therefore, the knowledge gained from this study can inform the strategic planning for diagnosing.

Economic evaluation is a general term for identifying, measuring, and valuing health interventions’ costs and consequences. Cost Effectiveness Analysis (CEA) of health interventions is one of these tools for determining the costs and consequences of interventions that can help healthcare decision-makers prioritize and allocate resources [16]. CEA is a useful tool to compare therapeutic interventions based on their costs and outcomes. In this analysis, outcomes are measured in a single unit, such as disease cases prevented or life years gained, and results are expressed in terms of the Incremental Cost Effectiveness Ratio (ICER) [17]. ICER is a ratio of the difference in cost to the difference in effectiveness of two methods. The higher the ICER value, the more money we must spend to get a unit of effectiveness, and the lower it is, the less money we must spend for an effective unit. Therefore, the method with a lower ICER value is more cost-effective [18].

This descriptive and analytical study was conducted to determine the most cost-effective COVID-19 diagnostic method for early treatment and prevention of disease transmission, given the importance of the issue for health sector policymakers and decision-makers.

## Methods

### Study design

This research was an economic evaluation of cost-effectiveness and descriptive-analytical type, in which chest CT, the combination of corona antibodies (IgM&IgG), and PCR methods for diagnosing COVID-19 patients among clinically suspicious cases were examined from the perspective of health economics. The hypothetical target population was patients with suspected COVID-19. First, using the available sources and evidence, the decision tree for the diagnostic strategies of COVID-19 is based on the scenarios designed according to the prevalence of COVID-19, with 5, 10, and 50% drawn separately [23]. Then, based on data on the percentage of disease prevalence, sensitivity, and specificity of the methods in diagnosing coronavirus patients and the costs of these strategies, the best solution was determined from the viewpoint of this software.

### Direct costs

The costs of methods diagnosing COVID-19, from the service provider’s perspective and in terms of dollars in 2021, were assessed in Iran. From this viewpoint, only direct medical costs (resulting from the time spent by the service provider, materials and equipment, and capital costs) were considered. With this method, the cost of a unit of CT, PCR, and coronavirus antibodies (IgM&IgG) was estimated, and indirect medical costs (which are caused by overhead costs) were not calculated.

### Effectiveness

In determining the effectiveness of the diagnostic strategies for COVID-19, the number of true positives, false positives, true negatives, and false negatives in a cohort of 1000 suspected patients with COVID-19 was used to determine the program’s effectiveness. To calculate the above, the prevalence, sensitivity, and specificity of each COVID-19 diagnostic strategy and the method suggested in Tamlyn Rautenberg’s study [19] were used, given in Table 1.

**Table 1:** Steps for determining the effectiveness of diagnostic strategies for COVID-19 using prevalence, sensitivity, and specificity

Step	Objective	Instruction	Formula
1	Define cohort	Insert a cohort of 1000	(A + B)+(C + D)
2	Find the total D+	Multiply the prevalence by the cohort to find the number of D + patients	(A + C)
3	Find the total D-	Subtract the D + from the total cohort to find D-	(B + D)
4	Find D + T+ (TP)	Multiply the sensitivity of the test to the total D + patients to get the D + T+	A
5	Find D + T- (FN)	Subtract the D + T + from the total D+	C
6	Find D-T- (TN)	Multiply the specificity of the test to the total D- patients to get the D-T-	D
7	Find D-T+ (FP)	Subtract the D-T- from the total D-	B
8	Find total T+	Add D + T + and D-T+	(A + B)
9	Find total T-	Add D + T- and D-T-	(C + D)

### Cost-effectiveness analytical model

The incremental cost-effectiveness ratio (ICER) was calculated according to the incremental cost required for the diagnosis of COVID-19 [20].

$$\text{I}\text{C}\text{E}\text{R}= \frac{{\text{C}\text{o}\text{s}\text{t}}_{\text{A}}-{\text{C}\text{o}\text{s}\text{t}}_{\text{B}}}{{\text{E}\text{f}\text{f}\text{e}\text{c}\text{t}\text{i}\text{v}\text{e}\text{n}\text{e}\text{s}\text{s}}_{\text{A}}-{\text{E}\text{f}\text{f}\text{e}\text{c}\text{t}\text{i}\text{v}\text{e}\text{n}\text{e}\text{s}\text{s}}_{\text{B}}}$$

${\text{C}\text{o}\text{s}\text{t}}_{\text{A}}$ and ${\text{C}\text{o}\text{s}\text{t}}_{\text{B}}$ are, respectively, the costs of strategies A and B, and ${\text{E}\text{f}\text{f}\text{e}\text{c}\text{t}\text{i}\text{v}\text{e}\text{n}\text{e}\text{s}\text{s}}_{\text{A}}$ and ${\text{E}\text{f}\text{f}\text{e}\text{c}\text{t}\text{i}\text{v}\text{e}\text{n}\text{e}\text{s}\text{s}}_{\text{B}}$ are, respectively, the effectiveness (true positive (TP), false negative (FN), true negative (TN), and false positive (FP)) of strategies A and B in the diagnosis of COVID-19 was considered. Subsequently, using the identified ICERs and comparing them with the cost-effectiveness threshold recommended by the World Health Organization for developing countries (one to three times GDP per capita), the cost-effectiveness of diagnostic strategies was assessed for COVID-19.

### Sensitivity analysis

After calculating the incremental cost-effectiveness ratio, the Tornado diagram was drawn to determine the sensitive parameters. Finally, a one-way sensitivity analysis was performed in terms of willingness to pay $7402 (the average value of willingness to pay per QALY gained in Iran [21]), and the robustness of the results was checked. The data obtained were analyzed using Treeage software.

## Results

**Fig. 1:**
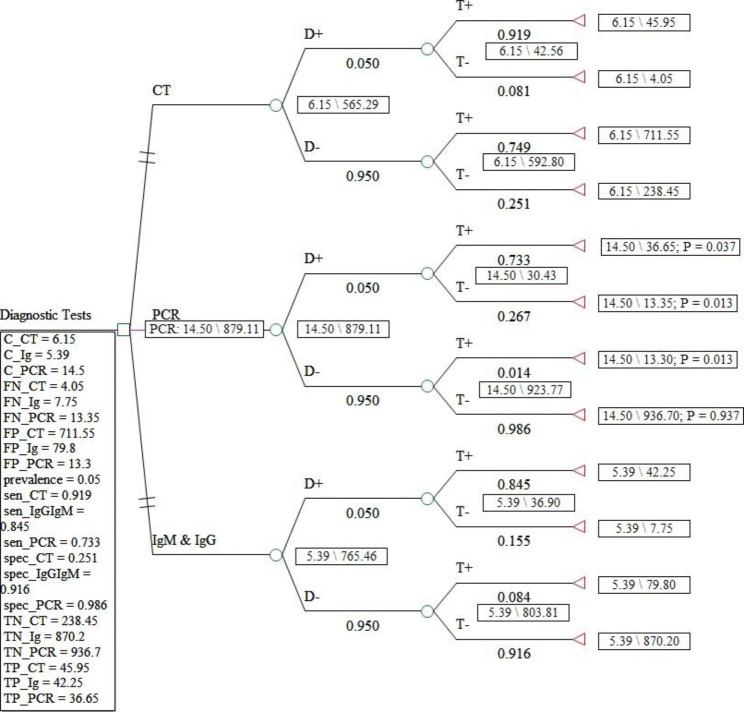
Decision tree model for the diagnostic strategies of COVID-19 in the scenario with a 5% prevalence

**Fig. 2:**
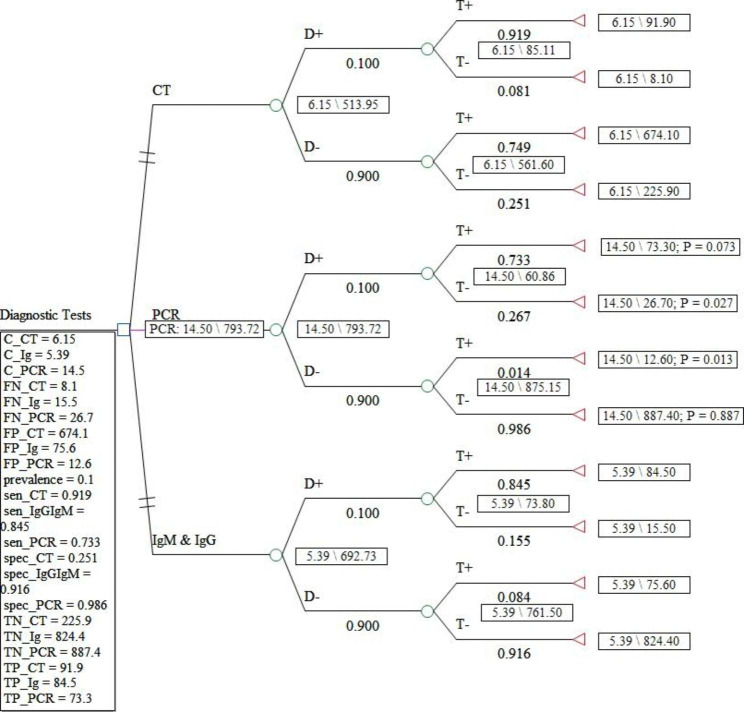
Decision tree model for the diagnostic strategies of COVID-19 in the scenario with a 10% prevalence

**Fig. 3:**
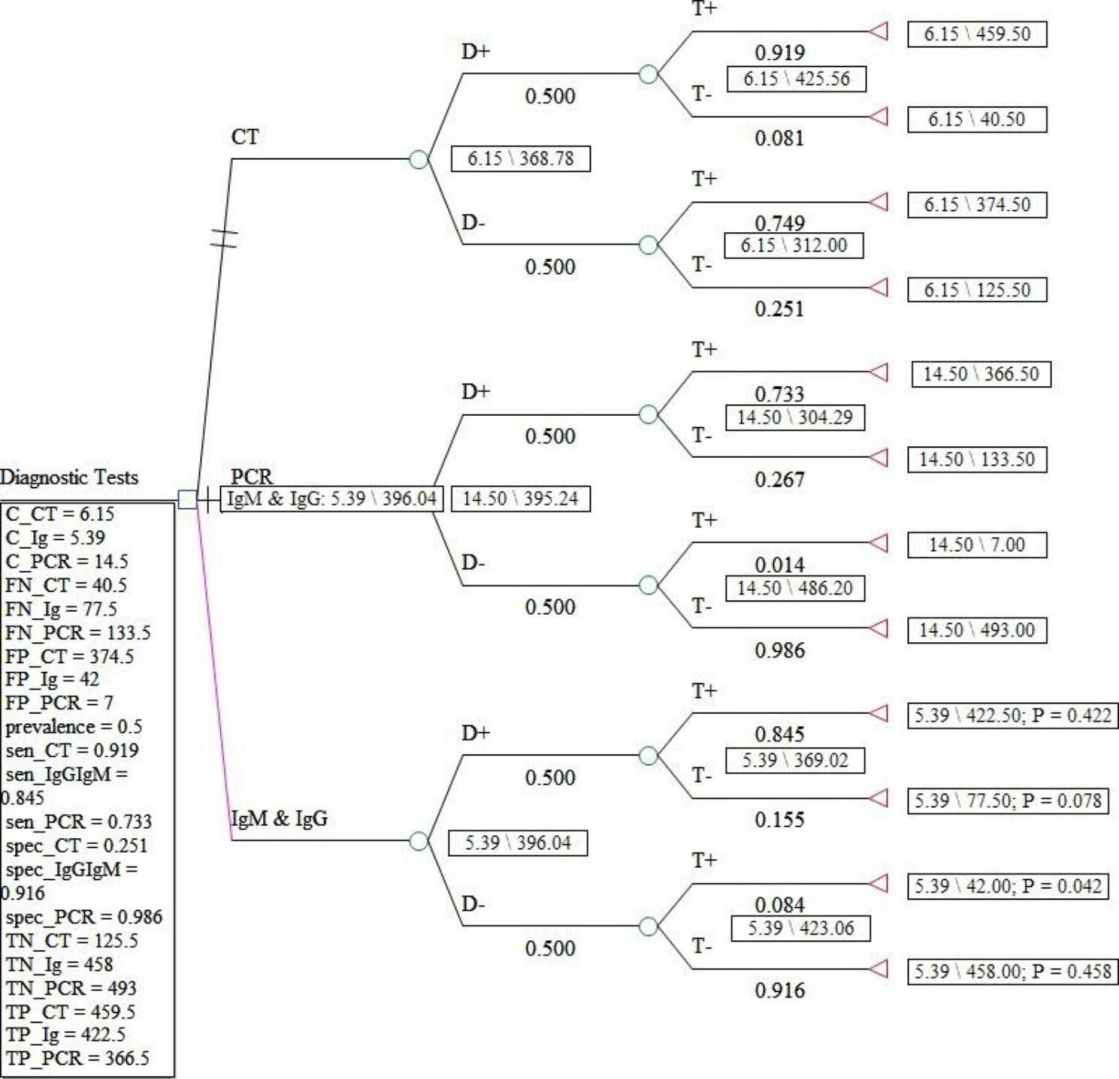
Decision tree model for the diagnostic strategies of COVID-19 in the scenario with a 50% prevalence

In Figs. 1 and 2, and 3, the decision tree for the diagnostic strategies of COVID-19 was drawn under three scenarios with 5, 10, and 50% prevalence. In these figures, the dominant and more cost-effective strategy in the scenario under study was shown in pink.

**Fig. 4:**
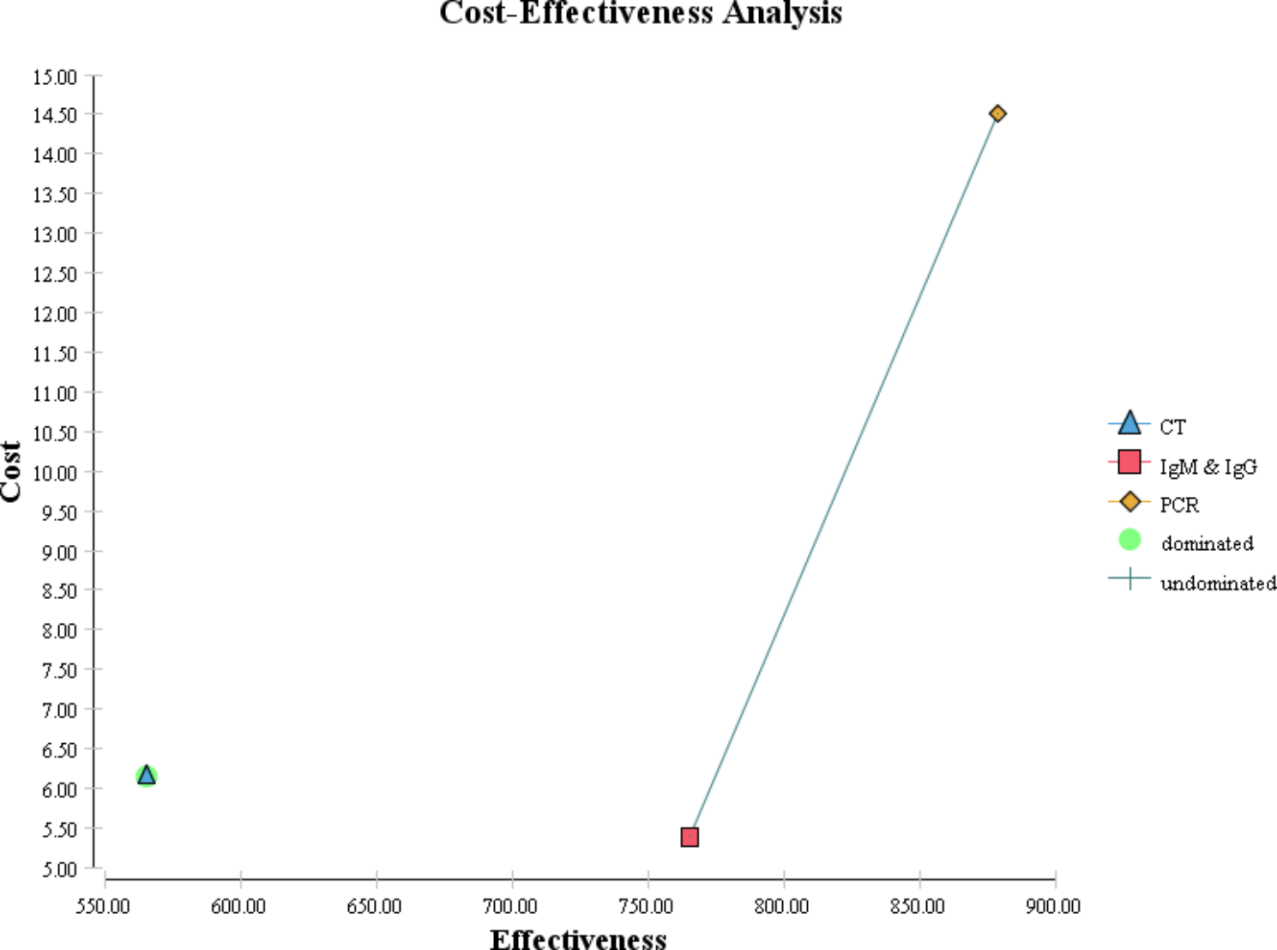
Cost-effectiveness map of diagnostic strategies for COVID-19 for 5% prevalence

**Fig. 5:**
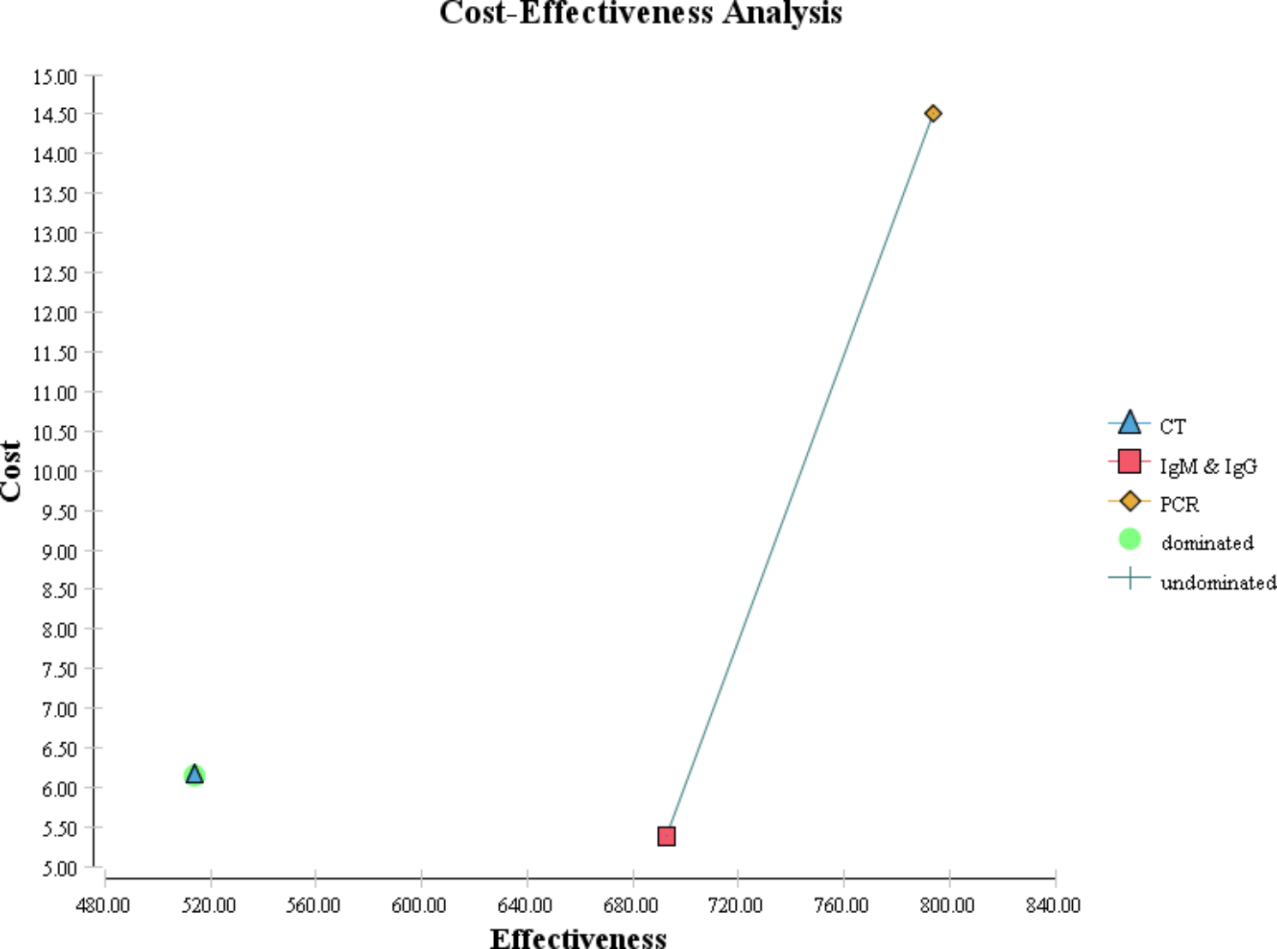
Cost-effectiveness map of diagnostic strategies for COVID-19 for 10% prevalence

**Fig. 6:**
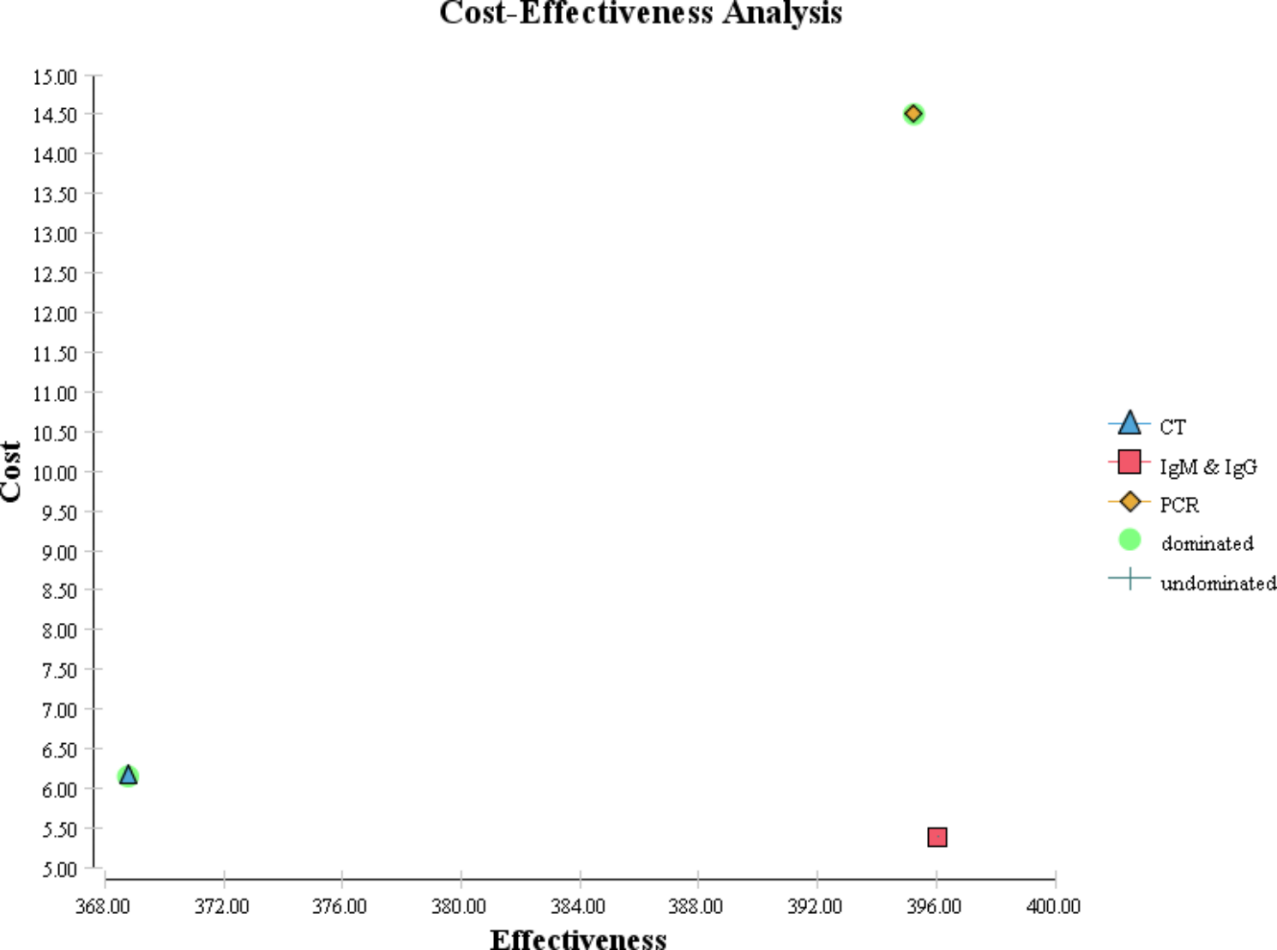
Cost-effectiveness map of diagnostic strategies for COVID-19 for 50% prevalence

Cost-effectiveness maps of COVID-19 diagnostic strategies for different scenarios are shown in Figs. 4 and 5, and 6. In the following figures, as it is known, the dominant strategies for the prevalence of 5, 10, and 50% of COVID-19 are PCR, PCR, and IgG&IgM, respectively.

To the estimate of ICER, calculation of expected costs and effectiveness of each COVID-19 diagnostic method is required after assigning the probabilities (prevalence, sensitivity, and specificity information) and the value of the outcomes (number of true positives, false positives, true negatives, and false negative) and the costs of each diagnostic strategy for each node through averaging out and folding back. The sensitivity and specificity of the CT, IgG&IgM, and PCR methods are (0.919 and 0.251), (0.733 and 0.986), and (0.845 and 0.916), respectively [22].

ICER was estimated based on the number of positive cases from the cohort of 1000 people suspected of COVID-19 and in terms of dollars for each diagnosed case of corona in the used diagnostic strategy. The summary of the output of the Treeage software for ICER under the three scenarios is given in Tables 2, 3 and 4.

**Table 2:** Cost-effectiveness analysis results from the decision tree model estimation for scenario 1 (COVID-19 prevalence: 5%)

Strategy	Effectiveness	Incremental Effectiveness	Cost	Incremental Cost	Incremental Cost Effectiveness	Average Cost Effectiveness	Subset
IgM & IgG	765.4612	0	5.39	0	0	0.007042	undominated
CT	565.2896	-200.172	6.15	0.76	-0.0038	0.010879	abs. dominated
PCR	879.1052	113.644	14.5	9.11	0.080163	0.016494	undominated

**Table 3:** Cost-effectiveness analysis results from the decision tree model estimation for scenario 2 (COVID-19 prevalence: 10%)

Strategy	Effectiveness	Incremental Effectiveness	Cost	Incremental Cost	Incremental Cost Effectiveness	Average Cost Effectiveness	Subset
IgM & IgG	692.7312	0	5.39	0	0	0.007781	undominated
CT	513.9528	-178.778	6.15	0.76	-0.00425	0.011966	abs. dominated
PCR	793.7233	100.9921	14.5	9.11	0.090205	0.018268	undominated

**Table 4:** Cost-effectiveness analysis results from the decision tree model estimation for scenario 3 (COVID-19 prevalence: 50%)

Strategy	Effectiveness	Incremental Effectiveness	Cost	Incremental Cost	Incremental Cost Effectiveness	Average Cost Effectiveness	Subset
IgM & IgG	396.0	0	5.39	0	0	0.014	Undominated
CT	368.8	-27.3	6.15	0.76	-0.028	0.017	abs. dominated
PCR	383.2	-12.9	14.5	9.11	-0.707	0.038	abs. dominated

Considering the per capita GDP of Iran and the threshold defined by WHO that if the ICER is less than three times the GDP per capita, the intervention is cost-effective, so in this study, in the scenarios with 5, 10, and 50% prevalence of COVID-19, respectively PCR, PCR, and IgG&IgM strategies are cost-effective.

Then, according to the Tornado diagram, one-way sensitivity analysis on the parameters that had the most significant effect on the incremental cost-effectiveness ratio was performed. These parameters include the specificity, sensitivity, and cost of IgG&IgM; cost, specificity, and sensitivity of CT; and the sensitivity and cost of PCR. The results of the one-way sensitivity analysis can be found in Table 5 for different scenarios of the spread of COVID-19. As can be seen, the results were not sensitive to the desired parameters.

**Table 5:** Results of one-way sensitivity analyses of selected parameters for different scenarios of the COVID-19 prevalence

Parameters	Base case	Range	Preferred strategy
Prevalence of 5%			
specifity of IgG&IgM	0.916	0.86 to 0.954	IgG&IgM-PCR
sensitivity of IgG&IgM	0.845	0.822 to 0.866	IgG&IgM-PCR
Cost of IgG&IgM	5.39	4.85 to 5.93	IgG&IgM-PCR
Cost of CT	6.15	5.53 to 6.76	IgG&IgM-PCR
specifity of CT	0.251	0.21 to 0.295	IgG&IgM-PCR
sensitivity of CT	0.919	0.898 to 0.937	IgG&IgM-PCR
sensitivity of PCR	0.733	0.681 to 0.78	IgG&IgM-PCR
Cost of PCR	14.5	13.05 to 15.95	IgG&IgM-PCR
Prevalence of 10%			
specifity of IgG&IgM	0.916	0.86 to 0.954	IgG&IgM-PCR
sensitivity of IgG&IgM	0.845	0.822 to 0.866	IgG&IgM-PCR
Cost of IgG&IgM	5.39	4.85 to 5.93	IgG&IgM-PCR
Cost of CT	6.15	5.53 to 6.76	IgG&IgM-PCR
specifity of CT	0.251	0.21 to 0.295	IgG&IgM-PCR
sensitivity of CT	0.919	0.898 to 0.937	IgG&IgM-PCR
sensitivity of PCR	0.733	0.681 to 0.78	IgG&IgM-PCR
Cost of PCR	14.5	13.05 to 15.95	IgG&IgM-PCR
Prevalence of 50%			
specifity of IgG&IgM	0.916	0.86 to 0.954	IgG&IgM
sensitivity of IgG&IgM	0.845	0.822 to 0.866	IgG&IgM
Cost of IgG&IgM	5.39	4.85 to 5.93	IgG&IgM
Cost of CT	6.15	5.53 to 6.76	IgG&IgM
specifity of CT	0.251	0.21 to 0.295	IgG&IgM
sensitivity of CT	0.919	0.898 to 0.937	IgG&IgM
sensitivity of PCR	0.733	0.681 to 0.78	IgG&IgM

## Discussion

In this study, according to the difference between the cost and performance of the diagnostic methods of COVID-19, cost-effectiveness analysis of these methods in several scenarios with different prevalences of the disease from 5 to 50% in a hypothetical cohort of 1000 patients suspected of coronavirus was examined. The most cost-effective strategy was determined by considering the costs and number of positive cases diagnosed based on the data of sensitivity and specificity of the diagnostic method in different scenarios.

The direct costs of COVID-19 diagnostic methods ranged from 5.39 to 14.5. Also, the expected effectiveness or the number of correctly diagnosed cases with coronavirus in a hypothetical cohort of 1000 people suspected of having COVID-19 in the scenario with a prevalence of 5% in the dominant diagnostic method, i.e., PCR, was 879 people, while this amount in the scenario of 10% in the dominant method, PCR, was 794 people. In the scenario of 50%, the dominant method, IgG&IgM, was equal to 396 people. According to the results of the incremental cost-effectiveness ratio obtained for different scenarios, for the prevalence of the disease between 5 and 10%, the PCR method was the most cost-effective test, but for conditions where the probability of the disease is estimated to be 50%, CT, and PCR are more expensive and less effective alternatives for correct diagnosis of coronavirus disease. Therefore, in this scenario, IgG&IgM was the dominant diagnostic method over CT and PCR.

The number of studies related to the economic evaluation of diagnostic methods for COVID-19 is limited. However, studies have recently been conducted for the economic evaluation of these methods. Machado de Asis et al. conducted a study to evaluate the cost-effectiveness of anti-SARS-CoV-2 antibody diagnostic tests for COVID-19 in Brazil. They compared eleven commercially available diagnostic tests from the perspective of the Brazilian integrated health system. They showed that lateral flow immunochromatographic assay (LFAs) tests are cost-effective for estimating low-prevalence of COVID-19, while ELISA assays are more cost-effective for high-prevalence scenarios [23]. Dolatshahi et al., in a systematic review of the economic evaluation of various laboratory diagnostic tests in the COVID-19 epidemic, concluded that despite its high cost, PCR was more effective than IgG in improving the quality of life and survival of patients with COVID-19; therefore, it is considered more cost-effective in some countries. The researchers suggested that more studies in low-income and developing countries are needed to evaluate the cost-effectiveness of PCR in the variable prevalence of infectious cases of COVID-19 [24]. According to the results of the study by Sriwijitalai and Wiwanitkit in the cost-benefit analysis of CT versus PCR for the diagnosis of COVID-19, the cost per benefit is higher for CT. This may mean that the PCR test is still a good choice for early detection of COVID-19 in clinically suspected cases, especially in developing countries with limited resources [25]. The results of the above-limited studies are almost consistent with the results of this study.

Despite the abundant resources to describe diagnostic decision analysis, the critical error of not including diagnostic test accuracy in the decision tree structure still needs to be addressed. These errors have implications for the accuracy of model results and, thus, affect decision-making. In this study, by applying basic epidemiological calculations (prevalence, sensitivity, and specificity) in the structure of the decision tree model in the form of visually attractive images, for the first time in the field of diagnosis, we attempted to overcome these errors, which is an advantage of this study.

Also, the results of this study using decision tree model estimates were robust to uncertainty (Table 4). The sensitivity analysis showed that changing the parameters to the maximum and minimum levels did not change the base case results. Additionally, cost sensitivity analysis showed that the cost of different interventions did not change the base case results.

Overall, implementation of the most cost-effective COVID-19 diagnostic method can eventually lead to financial savings for families and the health system by accurately diagnosing suspected cases and preventing the spread of infection due to early diagnosis of the disease.

In this study, there were limitations in access to data on the sensitivity and specificity of diagnostic methods. Additionally, the costs of these methods were calculated from the perspective of the Iranian healthcare system, so it is not easy to generalize the study results because of the different costs in the healthcare system of different countries.

The cost-effectiveness of COVID-19 diagnostic methods, in addition to their speed and accuracy, is a significant concern and provides evidence-based solutions for decision-making. In this sense, practical diagnostic tests are needed as an effective public health strategy. Therefore, in different health systems, due to the lack of resources, different strategies to improve diagnostic access to individuals with suspected coronavirus should be analyzed, and the most cost-effective identified.

## Conclusion

Based on the results obtained from this research, PCR tests are more cost-effective compared with other diagnostic methods for low prevalence of Covid-19, while IgM&IgG tests are more cost-effective for high estimated prevalence. The results of this study can help health managers in the field of decision-making and planning for a more optimal allocation of financial resources regarding the use of the most cost-effective diagnostic method for COVID-19, considering the epidemiological information of the disease.

## Data Availability

All data generated or analyzed during this study are included in this published article.
